# Characteristics of Patient Portals Developed in the Context of Health Information Exchanges: Early Policy Effects of Incentives in the Meaningful Use Program in the United States

**DOI:** 10.2196/jmir.3698

**Published:** 2014-11-21

**Authors:** Terese Otte-Trojel, Antoinette de Bont, Joris van de Klundert, Thomas G Rundall

**Affiliations:** ^1^Health Services Management & OrganizationInstitute of Health Policy & ManagementErasmus University RotterdamRotterdamNetherlands; ^2^Health Care GovernanceInstitute of Health Policy & ManagementErasmus University RotterdamRotterdamNetherlands; ^3^Health Policy & ManagementSchool of Public HealthUniversity of California, BerkeleyBerkeley, CAUnited States

**Keywords:** medical informatics, meaningful use, electronic health records, patient-centered care

## Abstract

**Background:**

In 2014, the Centers for Medicare & Medicaid Services in the United States launched the second stage of its Electronic Health Record (EHR) Incentive Program, providing financial incentives to providers to meaningfully use their electronic health records to engage patients online. Patient portals are electronic means to engage patients by enabling secure access to personal medical records, communication with providers, various self-management tools, and administrative functionalities. Outcomes of patient portals have mainly been reported in large integrated health systems. This may now change as the EHR Incentive Program enables and supports the use of patient portals in other types of health systems. In this paper, we focus on Health Information Exchanges (HIE): entities that facilitate data exchange within networks of independent providers.

**Objective:**

In response to the EHR Incentive Program, some Health Information Exchanges in the United States are developing patient portals and offering them to their network of providers. Such patient portals hold high value for patients, especially in fragmented health system contexts, due to the portals’ ability to integrate health information from an array of providers and give patients one access point to this information. Our aim was to report on the early effects of the EHR incentives on patient portal development by HIEs. Specifically, we describe the characteristics of these portals, identify factors affecting adoption by providers during the 2013-2014 time frame, and consider what may be the primary drivers of providers’ adoption of patient portals in the future.

**Methods:**

We identified four HIEs that were developing patient portals as of spring 2014. We collected relevant documents and conducted interviews with six HIE leaders as well as two providers that were implementing the portals in their practices. We performed content analysis on these data to extract information pertinent to our study objectives.

**Results:**

Our findings suggest that there are two primary types of patient portals available to providers in HIEs: (1) portals linked to EHRs of individual providers or health systems and (2) HIE-sponsored portals that link information from multiple providers’ EHRs. The decision of providers in the HIEs to adopt either one of these portals appears to be a trade-off between functionality, connectivity, and cost. Our findings also suggest that while the EHR Incentive Program is influencing these decisions, it may not be enough to drive adoption. Rather, patient demand for access to patient portals will be necessary to achieve widespread portal adoption and realization of potential benefits.

**Conclusions:**

Optimizing patient value should be the main principle underlying policies intending to increase online patient engagement in the third stage of the EHR Incentive Program. We propose a number of features for the EHR Incentive Program that will enhance patient value and thereby support the growth and sustainability of patient portals provided by Health Information Exchanges.

## Introduction

### The Meaningful Use Program

Passed into legislation in 2009, the Health Information Technology for Economic and Clinical Health (HITECH) Act included US $30 billion for accelerating and mainstreaming the use of health information technology [1]. The Act incentivizes the adoption of electronic health records (EHRs) by hospitals and physician practices and sets targets for the “meaningful use” of these EHRs to significantly improve patient care [2]. The EHR Incentive Program, also known as the Meaningful Use (MU) program, is a cornerstone of the HITECH Act. The program, which is regulated by the National Coordinator for Health Information Technology (ONC) and administered by the Center for Medicare & Medicaid Services (CMS), authorizes incentive payments through the Medicare and Medicaid Programs [3]. Through three stages, the program rewards providers that meaningfully use ONC-certified EHRs by meeting designated objectives and levies a financial penalty on those providers that fail to meet the objectives [4].

Stage 1 of MU, which was implemented in 2011, provided incentives for eligible physicians and hospitals to adopt EHRs with basic functionalities, such as capturing data electronically and exchanging information among care providers [5]. Subsequent to Stage 1 implementation, the number of providers meeting MU1 criteria with their EHRs sharply increased [6,7]. Up from 4% in 2010, 42% of hospitals surveyed in the 2012 American Hospital Association Health Information Technology Supplement fulfilled core Stage 1 requirements [8,9]. Further, it was estimated that by May 2012, 12.2% of US office-based physicians had successfully restructured their IT systems and practices to meet Stage 1 requirements [10,11].

Stage 2 of MU went into effect in 2014 for providers that demonstrated Stage 1 MU in 2011 [12]. In addition to using EHRs for continuous data capture and enhanced interprofessional information exchange, the second stage of the program emphasizes building online patient engagement capabilities on top of these EHRs [13,14]. One core objective specific to online patient engagement is that providers give at least 50% of their patient population the ability to view online, download, and transmit their health information within 4 days of the office visit (for physicians) and 36 hours of discharge (for hospitals). Of these patients, the provider must attest that 5% actually access their records online to view, download, or transmit information. Providers must also be able to securely message their patients and provide patient-specific educational resources to at least 10% of patients after office visits [15].

### Patient Portals in Light of Meaningful Use

Patient portals are vehicles for meeting these particular MU2 objectives by enabling secure messaging with health care providers and by giving patients access to their personal health records (PHRs) [16,17]. A PHR is a patient-centered tool used for managing health information and engaging in health promotion and management. The individual patients control their own PHR and may also insert information into the record that is not contained in an EHR [18]. A patient portal that is linked to a provider’s EHR is called a tethered patient portal [19]. Early evaluations of tethered patient portals suggest that they can improve chronic disease management, patient adherence to medications and preventive services, patient-provider communication, patient empowerment, and patient satisfaction [20]. These outcomes have so far been reported from portals within large and integrated delivery systems, such as Kaiser Permanente and the Veterans Affairs health system, with system-wide EHRs. Through a combination of their comprehensive coverage of a defined eligible population and EHR integration, these portals give patients one access point through which they can view their information and interact with all of their providers in the system. Due to this comprehensiveness and integration, these portals can trigger mechanisms such as enhanced patient insight into their complete health information, interpersonal continuity of care, and patient convenience, which are instrumental to achieving the outcomes listed above [21].

However, as MU2 introduces financial incentives for online patient engagement, the program is now enabling and stimulating the development of patient portals in health system contexts other than these large integrated delivery systems, including Health Information Exchanges (HIEs).

### Patient Portals in the Context of Health Information Exchanges

Regional or statewide HIEs facilitate information transfer among participating hospitals and physicians’ independent and non-interoperable EHRs. In 2013, 90 community-based and 45 statewide HIEs were reported in the United States [22]. By facilitating information transfer among independently operating clinicians, laboratories, hospitals, pharmacies, and health plans, these HIEs play an important role in connecting providers in fragmented contexts [23]. According to a Robert Wood Johnson Foundation report [8], by the end of 2012, 30% of US hospitals and 10% of ambulatory practices sent and received data through HIE efforts. This number has increased significantly over the last years, up from 14% and 3% in 2010 for hospitals and ambulatory practices respectively [8]. With information transfer being an ever more crucial component of the ONC’s agenda, many HIEs will continue to receive start-up grants, demonstration project grants, and ongoing operational support from the Department of Health and Human Services [24].

As health service providers that participate in these HIEs now seek ways to capture the MU2 incentives, some HIEs will play an increasing role in developing patient portals. Such HIE-sponsored patient portals may enable online patient engagement opportunities not previously seen outside of integrated health delivery systems. By consolidating information, which is typically spread across a range of independent providers’ EHRs, patient portals that are developed by HIEs in fragmented contexts can give patients an overview of their health information. Since some patients—especially patients with complex conditions—see multiple providers [25], this single access point alleviates for patients the hassle of accessing their records and using other health care services through different portals, each with their own passwords, usernames, and interfaces. By achieving comprehensiveness and integration, patient portals in these systems may create patient value comparable to that observed in integrated systems, and thereby trigger outcomes similar to those reported from these systems. As pointed out by Otte-Trojel et al (2014) the patient value of portals that develop in fragmented systems may even exceed that of portals in integrated systems by breaking down siloes in fragmented provider-centric systems [21].

The achievement of this value from HIE-sponsored portals is challenged by the reality that only 10-30% of providers are currently linked to an HIE. However, as HIE participation rates grow each year, the coverage of HIE-sponsored portals will likely increase at a proportional rate.

### Study Aim

The potential of HIE-sponsored patient portals to deliver patient value in fragmented health system contexts is significant, especially for patients who see multiple providers. Yet, realizing this potential depends on physicians and hospitals that are members of the HIEs adopting the portals into their practices. The aim of this study is to report on the development and rollout of the first HIE-sponsored portals and to explore the early effects of the MU incentives on these developments. Specifically, we examined the following questions: (1) What are the characteristics of early-stage HIE-sponsored portals?; (2) What are the major factors affecting providers’ adoption of HIE-sponsored portals?; and (3) What factors will drive the further development and adoption of patient portals in the HIE context?

## Methods

Based on the list of HIEs in the 2013 eHealth Initiative report [22], we identified the HIEs that on their websites announced that they were either developing or offering a patient portal. As of early 2014, such activity was seen in HIEs in Pennsylvania, Kansas, California, and Texas. One of these patient portals has been implemented in a small set of provider practices, one is currently being rolled out in a few practices, and two are still in the piloting phase. Although being a relatively small sample, the four HIEs represent the HIEs that are developing patient portals at present and provide useful experience and information for other HIEs that intend to develop portals as well as for policy makers interested in the effects of MU criteria and incentives on patient portal development. Key characteristics of the four front running HIEs are presented in Table 1.

We contacted the directors of these four HIEs in February and March 2014, and all directors agreed to participate in our study. We asked all four HIE directors to refer us to other relevant HIE staff or providers that were in the process of implementing the portals in their practices. We collected data in March and April 2014. As part of our data collection, we obtained documents and conducted 10 in-depth interviews with 8 people, including 4 HIE directors, 2 HIE project managers from two different HIEs, and 2 providers participating in one of these HIEs. The 2 providers were from the HIE that had implemented the patient portal in a small set of provider practices at the time of the data collection. In addition, the HIEs provided us with relevant documents, including presentations, training and installation guides, and annual reports. We conducted content analysis [26,27] of the interview transcripts and acquired documents to extract information relevant to our research questions.

**Table 1:** Characteristics of HIEs implementing patient portals.

	Kansas Health Information Network (KHIN)	Healthcare Access San Antonio (HASA)	Keystone Health Information Exchange (KeyHIE)^a^	Santa Cruz Health Information Exchange
Date founded	2010	2005	2005	1998
Service area	Statewide	22 counties around San Antonio	53 counties in Pennsylvania	Santa Cruz county
Penetration	6000+ providers, 700+ organizations	683,000 providers, 29 organizations	1,500 providers, 40 organizations	400 providers, 200 organizations
Unique patients	2+ million	860,000	3,7 million	300,000
Patient portal name	MyKSHealth eRecords (previously My Health eRecord)	MyHASA	MyKeyCare	Santa Cruz HIE patient portal
Patient portal vendor	NoMoreClipboard	Mana Health	Get Real Health	NoMoreClipboard
Current patient portal users			2400+	

^a^Only KeyHIE had active patient portal users at the time of data collection in spring 2014.

## Results

### Characteristics of Early-Stage Patient Portals Sponsored by Health Information Exchanges

To meet the MU2 requirements, the portals that are currently being developed by HIEs focus on enabling patients to view and download summary of care documents and transmit them to other providers. The HIE-sponsored portals are untethered, since they are not directly linked to an EHR. For two of the HIEs, data will be transferred to each patient’s PHR through the ONC’s secure emailing system, *Direct*. As their technical capabilities expand, the plan is to enable automatic population of the PHRs with data, including summary of care documents, lab results, immunization reports, etc. The other two HIEs already have such automatic transfer capacities, meaning that data can be pulled directly into the PHR. The HIEs have set up procedures to allow providers to flag information in their EHRs that they would not like to share with patients or that they would prefer to discuss with patients before making accessible.

At this early stage of development, patients will be able to store documents and enter information (eg, about their diet or exercise) in their PHRs, but as the portals evolve, the plan is to also enable transmission of such patient-generated information to relevant health care providers. To comply with MU, secure messaging between patients and providers is a standard feature in all the portals.

In addition, individual providers may choose to offer EHR-linked features, such as appointment scheduling, bill payment, patient preregistration, and prescription refill through the portals. In fact, one of the interviewed providers mentioned that they were discussing ways to add a scheduling component and a bill payment component to the portal. However, since the portals are untethered, this will require the providers to install such applications at their end and subsequently integrate these applications with the patient portal. As several HIE directors and project managers explained, incorporating such features into the portal would require extensive collaboration between the provider and the HIE to achieve the necessary technical and workflow integration. Requiring far less integration work, at least two of the HIEs are also planning to provide non–EHR-linked features through the portals, such as various educational, self-management, and wellness applications.

All four portals will be accessible through both the websites of participating providers and a central HIE website. The providers may choose to brand the portal to fit their organization, in which case the portal’s interface may differ across these providers. One of the HIEs aired intentions to eventually also make its portal available on mobile devices, such as mobile phones and iPads.

All of the HIEs are planning to incorporate the cost of their portal into the overall HIE provider participation fee, while at least one is considering assigning some of the cost to patients in the form of a US $3-5 monthly service fee. That HIE had conducted several focus group interviews with patients in the region to learn about their willingness to pay and had concluded that this reimbursement model was feasible. A number of studies have examined patients’ willingness to pay for access to patient portals. Results from these studies indicate that 40-70% of patients with Internet access would be willing to pay a small fee for use of standard patient portal features such as viewing their medical records and secure emailing with their providers [28,29].

While all the HIEs see vast potential for their patient portals to improve patient health and provider workflow in their state or region, at the time of the interviews none of the HIEs had set specific targets on these areas. The critical milestone for all four HIEs is to be able to attest MU by the start of their providers’ reporting periods in either July or October 2014. Afterwards, the most important success factor is to attain a certain level of provider adoption and patient use of their portals.

### Factors Affecting Providers’ Adoption of Portals Sponsored by Health Information Exchanges

#### Overview

The main issue limiting HIE-participating providers’ uptake of HIE-sponsored portals is the difficulty HIE leaders have in convincing providers that the value of the HIE portal exceeds that of the EHR-tethered portal that most providers are offered by their EHR vendor. As a result of this competition from the EHR vendors’ portals, an HIE-participating provider’s decision to adopt the HIE-sponsored portal seems to largely be a trade-off between functionality, connectivity, and cost.

#### Functionality

From our interviews with HIE directors and managers, we identified three main challenges that currently limit the HIE-sponsored portals’ degree of functionality. It should be noted that the reported significance of these challenges varies across the four HIEs.

First, the missing linkage between providers’ EHRs and the HIE limits the capacities of the HIE-sponsored portals compared to their tethered counterparts in several ways. As noted above, in this early stage, EHR-linked features of convenience for patients (such as appointment scheduling) may not be available through the HIE-sponsored portals. Until full integration between providers’ EHR and the HIE-sponsored portal is realized, one HIE director noted that a solution could be to simply refer patients to relevant providers’ EHR features through the portal. However, accessing such features would require patients to use a separate login for each individual provider feature, thus contradicting the rationale behind a shared portal. Furthermore, for the HIEs that are still working on enabling automatic flow of information from the HIE to the patients’ records, providers or their administrative staff will have to manually perform this step by sending relevant documents via an email function. While this may be feasible in small practices with small patient volumes, for bigger practices, manually transferring data after each patient visit or discharge is an unsustainable solution.

Second, all the HIEs are challenged by creating the reporting formats required for providers to attest MU. This challenge is augmented by the fact that the HIEs are not tied to the providers’ EHRs, which contain administrative data on patient contacts such as when an office visit or discharge took place. Such information is necessary, for example, to generate reports certifying that information was made available to patients in a timely manner. According to the HIEs, no perfect solution is in place yet. For now, attesting MU2 requires that the providers themselves link the data generated by the HIE with information in their own EHR. Since clinical documents are generated through the EHR, many providers have turned to their EHR vendors for advice on meeting MU2. Since these vendors could guarantee only that providers would meet the requirements through the vendors’ portals, directors and project managers in two HIEs reported that many providers in their network came to believe that their EHR vendor’s tethered portal was a better option than the HIE-sponsored portal.

Third, to varying degrees, the HIEs have yet to fully resolve issues regarding patient matching [23]. When data are exchanged among providers in a given HIE network, data from different providers are matched to a given patient using probabilistic matching algorithms [30]. If available, these algorithms take into account various patient identifiers, such as name, gender, date of birth, social security number, address, and phone number. In fact, one HIE uses 17 patient identifier options for matching. Since the data are being sent only to providers, the threshold for what is considered an adequate match for data with a patient is less than 100% and varies by HIE. When in doubt the providers are able to double-check the data with the patient at the time of a patient visit. However, this situation is different when it comes to populating PHRs, which can be accessed by patients via their portals. Ideally there should be a 100% match of patient identifiers so that patients never receive incorrect data in their PHR. In cases where it is not possible to achieve a match, data cannot be sent to the PHR. This challenge is especially prominent if there is considerable variation in how participating providers’ EHRs format names and addresses, in the quality of data entered in the system at the point of patient registration, and if there are duplicate records for the same patient [31]. Providers with EHR-tethered portals do not share the same challenge, since they can always achieve a full match based on patients’ medical record numbers generated within their systems.

#### Connectivity

The core value of HIE-sponsored portals is their connectivity. According to the MU specifications, to meet the 5% target of patients that view, download, or transmit their information, a provider must have contributed some of the information to the shared portal, but not necessarily the particular information that was viewed, downloaded, or transmitted by the patient. There is consensus among the interviewees that the network externality of sharing credit for patient contacts and thereby collaborating to reach the 5% target is the main selling point of the HIE-sponsored portals to the HIE member provider organizations. As the directors and managers in all four HIEs explained, for many clinics and hospitals this shared incentive makes it more feasible to achieve the 5% target through the shared HIE-sponsored portal compared to having their patient population view records generated only in their respective office, clinic, or hospital. A manager from one medical center pointed out that a positive consequence of this incentive mechanism could be that providers will encourage each other to adopt the HIE-sponsored portal.

#### Cost

A notable advantage of the HIE-sponsored portals is lower costs to providers of acquiring a portal. As explained earlier, most HIEs plan to roll the portal costs into the overall HIE participation fee, regardless of whether a provider actively uses the portal. Smaller practices are especially sensitive to the high cost of EHR-tethered portals and may prefer this more affordable option. Moreover, one HIE director pointed out that the shared portals can operate with a lower overhead, since providers can share certain functions, such as a helpdesk to register patients and respond to patient inquiries.

Nevertheless, this cost incentive is not available for providers that have already implemented a patient portal as part of their EHR. Due to the ONC’s certification of “complete EHRs” that include patient portals, many providers have already implemented an EHR-tethered portal. The “complete EHR” certification has meant that EHR vendors can guarantee only that their EHRs meet MU requirements if a patient portal is included in the package. For providers who have purchased a patient portal as part of their EHR package, the added value of participating in an HIE-sponsored portal would have to compensate for the (sunk) cost of having an already-purchased portal remain unused (one might note that if the cost of the HIE-sponsored portal is included in the overall participation fee, the expense on a non-used HIE-sponsored portal could also be considered a waste). As one project manager expressed: “MU is kind of the best and the worst of worlds at the same time”.

### Factors Likely to Impact the Further Development and Adoption of Portals Sponsored by Health Information Exchanges

As outlined above, for HIE-participating providers that are considering implementing a patient portal, their decision to adopt a HIE-sponsored portal may largely be a trade-off between functionality, connectivity, and cost. Yet, according to the interviewed HIE directors and managers, the widespread uptake of patient portals is also inhibited by a general lack of provider interest in sharing information and communicating with patients online. The HIE directors and managers noted that a considerable share of their members express concerns with respect to patient portals, with the main points of concern being that secure messaging will lead to a boost in patient contacts and that patients will not able to interpret or cope with the clinical data in their records. It is worth noting that the literature provides a mixed account of secure messaging’s effect on health service utilization. Some studies show that secure emails increase utilization of hospitalizations, in-person visits, and telephone contacts [32-35], while one has demonstrated the opposite [36]. There are no good estimates of the proportion of providers that are interested in online patient engagement, and this proportion may vary by state and county. However, the perception among the HIE directors and managers that we interviewed is that a large proportion of providers in their network are not highly motivated by the MU incentives. This perception is supported by reports of the uptake of MU2 requirements. By June 2014, only eight eligible hospitals and 447 eligible professionals had attested to Stage 2 meaningful use [37,38]. Thus, although the HIEs develop patient portals to help their members comply with MU2, this financial incentive may not be enough to guarantee widespread uptake among providers.

Nonetheless, the notion among the HIEs is that as more and more providers include a patient portal in their service portfolio, patients will start demanding that their non-compliant providers offer similar capabilities for online engagement. If the HIEs’ predictions about such network externalities hold true, the MU financial incentives may encourage HIEs to provide the infrastructure for patient portals, but ultimately the patients will have to drive the widespread incorporation of the portals into their providers’ practices. Most patients recognize the benefits of patient portals: in a 2011 nationwide survey, 70% of patients indicated that they would want to access portals with comprehensive PHRs if they were made available to them [39].

## Discussion

### Principal Findings

MU2 incentives have ignited interest among some health service providers in fragmented systems to install or further develop patient portals. Such arrangements can be made via EHR-tethered portals within individual provider practices or via portals shared by the HIE network. While both arrangements enable online patient engagement, the latter may lead to more patient value by simulating the connectivity of portals in larger and integrated systems from which outcomes have mainly been reported thus far. Indeed, conversely, development of patient portals solely at the individual provider level could result in sustained fragmentation of patient information. As explained earlier, this fragmentation may have the most serious implications for patients receiving care from multiple providers, who would have to access several patient portals to view all their personal health information and interact with all their providers. However, the functionality of early stage HIE-sponsored portals may be lower than EHR-tethered portals, limiting their adoption. As we have explained, the MU incentives reward connectivity by allowing patient contacts to count towards all providers that contribute to a shared HIE-sponsored portal. Yet, it is still too early to conclude whether this incentive mechanism will be enough to ensure provider adoption of shared portals over individual portals. However, according to our informants, due to a general disinterest among providers to engage their patients online, financial incentives alone may not be enough to drive the widespread adoption of patient portals. Instead, by making online engagement tools a market differentiator, patients themselves may ultimately be the driving force behind patient portal adoption.

Hence, a focus on patient value will be imperative to drive the development of patient portals, and more importantly, to realize the outcomes possible through the use of portals [40]. If the patient portals do not deliver sufficient functionality and meaning to patients, they will likely not be able to generate traction among patients, caregivers, and health care consumers to create the necessary demand. Further, if the portals do not adequately engage patients, patients may not capitalize on their online capabilities to spur quality, efficiency, and patient safety outcomes envisioned in the EHR Incentive Program [41]. Patient engagement is dependent on patients’ perceptions that portal services enrich their current care and patient-provider relationship [42,43]. Thus, identifying ways to optimize and embed patient-valued portal capacities and functionalities into their health care services is crucial to achieving desired outcomes, since achieving these outcomes relies on patients to co-produce the outcomes by appropriately using the services [44]. We argue that portals that connect with multiple providers to give patients only one highly connected portal have the highest value to patient. The improvement of certain process and health outcomes is especially critical for patients with complex or chronic conditions who receive care from multiple providers and who account for an increasingly large part of the burden of disease [45]. Indeed, these patients will likely benefit most from a shared portal that allows them to interact and access information from their entire network of providers.

### Limitations

This study has a number of limitations. First, our principal findings and policy recommendation are based on experiences of a small number of HIEs. However, these HIEs represent the first ones developing patient portals to take part in MU in early 2014. Second, only early results from MU implementation on HIE portal development are assessed at this time. In fact, only one of the HIEs had a fully operational portal at the time of study, whereas the other three were in the implementation or pilot phases. Thus, a follow-up study 2-3 years from now would be relevant to understand the longer-term effects of the MU program on patient portal development, adoption, and use. Third, since we do not have information from patients, our results are constrained to the perceptions of the barriers and facilitators of patient portal development from HIE manager’s perspective. A follow-up study could benefit from including patient users and non-users as research subjects.

### Policy Recommendations

Due to the importance of patient interest and engagement for the success of the program, an important question is whether the prevailing patient portal development that emphasizes provider-centric benefits will lead to portals that rouse the necessary patient demand. We argue that optimizing patient value should be a prime principle underlying efforts to promote online patient engagement in the third stage of the MU program, which is set to start in 2017. The Stage 3 program specifications are now under development, informed by the experience of 12 projects funded by the Agency for Healthcare Research and Quality that will propose relevant revisions to the program’s overall objectives and specific measures [46]. In the following, we point to some MU Stage 3 program features identified in our research that could enhance the feasibility and success of HIE-sponsored portals, including Stage 3 financial incentives, guidelines, and technical requirements.

Shared portals can be promoted through financial incentives that further reward connectivity by strengthening incentives to portals that cross multiple providers. According to one HIE director, “MU3 ought to focus on giving the patients just one portal”. The degree of connectivity will also benefit from financial initiatives aimed at increasing the overall rates of HIE participation; as noted earlier, the potential connectivity possible through HIE-sponsored portals may be limited unless HIE participation rates increase. Furthermore, similar incentives could be targeted towards establishing links with other data repositories, particularly the federal Blue Button [47]. Developed as part of the ONC’s Standard and Interoperability framework, Blue Button gives, among others, veterans and Medicare beneficiaries access to their electronic records and the ability to transmit them to other providers or family members [48]. A link with Blue Button may enhance the value of portals by providing a critical mass of data and thus higher connectivity of patient information in a state or region.

Guidelines and other technical and organizational support mechanisms could assist other organizations and networks in developing shared, untethered portals and thereby mitigate some of the challenges faced by the pioneering HIEs. Specifically, the existing ONC specifications for reporting MU poorly match the HIE situation, and further guidance on how to create accurate reporting formats could facilitate this process. Similarly, solving issues around patient matching could also increase the likelihood that providers will adopt HIE-sponsored portals. More generally, such initiatives can be supported by the creation of an HIE collaborative, specifically aimed at disseminating and exchanging successful innovations from HIEs that are developing and implementing patient portals.

Technical requirements could focus on features and capabilities that enhance the functionality of untethered portals to patients. In addition to promoting further integration with providers’ EHRs, this could entail giving patients access to more sections of their medical record or more options to interact with providers. Moreover, but likely further down the line, portals could leverage mobile technology to allow for integration with various wellness and health management applications that could further personalize the portal services. On a more urgent note, the ONC’s proposed certification requirements for 2015 [49], which involve revoking the “complete EHR” certification in favor of a more modular approach, should be put into force to level the playing field between tethered and untethered portals.

Finally, on a broader level, in consultation with patient representatives, the ONC, CMS, and the patient portal-developing HIEs could engage in a dialogue to define realistic targets for developments and outcomes of the HIE-sponsored portals and weave time-specific goals into forthcoming ONC/CMS strategies.

## Abbreviations

CMS: Center for Medicare & Medicaid Services
EHR: electronic health record
HASA: Healthcare Access San Antonio
HIEs: Health Information Exchange
HITECH: Health Information Technology for Economic and Clinical Health
KeyHIE: Keystone Health Information Exchange
KHIN: Kansas Health Information Network
MU: meaningful use
ONC: National Coordinator for Health Information Technology
PHR: personal health record
